# Preoperative pain hypersensitivity is associated with axial pain after posterior cervical spinal surgeries in degenerative cervical myelopathy patients: a preliminary resting-state fMRI study

**DOI:** 10.1186/s13244-022-01332-2

**Published:** 2023-01-24

**Authors:** Qian Su, Jie Li, Xu Chu, Rui Zhao

**Affiliations:** 1 Department of Molecular Imaging and Nuclear Medicine, Tianjin Medical University Cancer Institute and Hospital, National Clinical Research Center for Cancer, Tianjin Key Laboratory of Cancer Prevention and Therapy, Tianjin’s Clinical Research Center for China, Tianjin, 300060 China; 2 Graduate School, Tianjin Medical University, Tianjin, 300203 China; 3 Department of Minimally Invasive Spine Surgery, Tianjin Hospital, Tianjin University, Tianjin, 300211 China; 4 Department of Orthopedics, Honghui Hospital, Xi’an Jiaotong University, Xi’an, China; 5 Department of Orthopedics Surgery, Tianjin Medical University General Hospital, Tianjin, 300052 China

**Keywords:** fMRI, Postoperative axial pain, Central sensitization, Support vector machine, Degenerative cervical myelopathy

## Abstract

**Objective:**

To test whether preoperative pain sensitivity is associated with the postoperative axial pain (PAP) in degenerative cervical myelopathy (DCM) and to explore its underlying brain mechanism.

**Methods:**

Clinical data and resting-state fMRI data of 62 DCM patients along with 60 age/gender matched healthy participants were collected and analysed. Voxel-wise amplitude of low frequency fluctuation (ALFF) was computed and compared between DCM patients and healthy controls. Correlation analyses were performed to reveal the association between the clinical metrics and brain alterations. Clinical data and ALFF were also compared between DCM patients with PAP and without PAP.

**Results:**

(1) Relative to healthy participants, DCM patients exhibited significantly lower preoperative pain threshold which is associated with the PAP intensity; (2) Relative to patients without PAP, PAP patients exhibited increased ALFF in mid-cingulate cortex (MCC) and lower preoperative pain threshold; (3) Further, multivariate pattern analysis revealed that MCC ALFF provide additional value for PAP vs. non-PAP classification.

**Conclusion:**

In conclusion, our findings suggest that preoperative pain hypersensitivity may be associated with postoperative axial pain in degenerative cervical myelopathy patients. This finding may inspire new therapeutic ideas for patients with preoperative axial pain.

**Supplementary Information:**

The online version contains supplementary material available at 10.1186/s13244-022-01332-2.

## Key points

- DCM patients exhibited pain hypersensitivity preoperatively compared to healthy participants.
- The pain hypersensitivity is associated with postoperative axial pain intensity in DCM.
- MCC ALFF could be used to predict occurrence of PAP in DCM.

**Supplementary Information:**

The online version contains supplementary material available at 10.1186/s13244-022-01332-2.

## Introduction

Degenerative cervical myelopathy (DCM), which characterised by degenerative changes in the cervical spine, is the most common cause of non-traumatic spinal cord injuries in adults, and requires timely surgical decompression to prevent progressive neurological deficits [1–3]. Until now, surgery remains the foremost treatment option for patients with DCM, and a corrective surgery at an early stage of DCM may effectively change the unfavorable prognosis for patients [4]. Despite that surgical strategy for DCM has been controversial (e.g., anterior approach or posterior approach), posterior laminoplasty and laminectomy still are the standard treatment for effective decompression of multi-level lesions, and their clinical efficacy remain satisfactory while the surgery-related complications are significantly fewer than anterior approaches [5, 6]. However, a major resulting complication—postoperative axial pain (PAP, i.e., pain from the nuchal to the periscapular region), has been largely overlooked and related factors remained controversial [7, 8]. Currently, there is no effective perioperative management to prevent or reduce this vexing complication and thus needs further investigation for its potential mechanism [8]. Recently Zheng et al. investigated the pressure pain thresholds, temporal summation and conditioned pain modulation in DCM patients and found that preoperative endogenous pain modulation deficiency may be associated with axial pain after posterior decompression surgery indicating preoperative pain hypersensitivity in DCM might contributed to the prevalence of PAP [9]. However, the brain mechanism underlying such phenomenon is still unknown.

In the past decade, resting-state fMRI (rs-fMRI) has been widely applied for investigating neural mechanism of pain. Researchers have highlighted the potential use of rs-fMRI data in interpreting the neuropathology and developing prognostic biomarkers for chronic pain [10–12]. Increasing evidence has uncovered structural and functional brain changes in regions associated with pain modulation, and such changes have been associated with pain intensity, disability, and pain sensitivity in patients with chronic pain [13–16]. In these studies, Amplitude of Low Frequency Fluctuation (ALFF), which is a widely used rs-fMRI metric, has gained much attention for its simplicity, interpretability and replicability among commonly used rs-fMRI metrics [17–19]. Moreover, recent studies have shown that ALFF was tightly associated with cerebral blood flow [20, 21] and task-evoked activation [22, 23] and could serve as a biomarker for predicting the analgesic-response in cervical spondylosis patients with chronic neck pain [17]. Therefore, ALFF is ideally suited for investigating PAP in DCM patients, considering the current lack of the knowledge for the underlying brain mechanism. Understanding such mechanism may be beneficial in enabling stratification in the perioperative period of DCM, and developing new analgetic strategy for reduce the PAP in DCM patients.

Therefore, in our current study, we conducted rs-fMRI to test whether preoperative pain sensitivity is associated with PAP in DCM patients and its association with brain alterations measured by ALFF; and to explore the utility of brain imaging markers based on ALFF for predicting the occurrence of PAP in DCM patients.

## Materials and methods

### Subjects

A local institutional review board approved this study, and all participants signed written informed consents. The detailed inclusion and exclusion criteria can also be found in Additional files (*Subjects’ inclusion criteria*). A total of 62 DCM patients and 60 Healthy Controls (HC) were recruited between 2015 and 2020.

### fMRI data acquisition and preprocessing

The detailed information of data acquisition and preprocessing steps can be found in Additional files (*fMRI data acquisition and preprocessing*).

## Clinical assessment

### Preoperative

Each DCM patient was evaluated using the Japanese Orthopaedic Association (JOA) score, which is the most widely used scale for determining the severity of DCM in clinical practice [24]. The Pain Vigilance and Awareness Questionnaire (PVAQ) was assessed in both DCM patients and healthy controls. The PVAQ is ranged from 0 (minimal attention to pain) to 80 with a higher score indicating more attention to pain [25]. Electrical stimulation was used preoperatively to determine the pain threshold in both DCM patients and healthy controls. Electric stimulation (0.2-ms square wave pulse; Digi-timer DS-7A, Hertfordshire, England) of the posterior neck area was then performed using a bipolar probe with the anode placed distally (20 mm inter-electrode distance). This stimulation method reduces the risk of peripheral sensitization and receptor fatigue. Stimulus intensities corresponding to the sensory detection and pain detection thresholds (pricking sensation) were registered using the method of ascending limits in 4 series (the first was discarded). Both DCM patients and healthy controls were instructed to immediately respond verbally when each level was felt. The perception of a pricking sensation is thought to correspond to Aδ-fiber activation. No rating scale was administered because only detection thresholds were assessed. Preoperative neck pain intensity was assessed using a standardised numerical rating scale (NRS) ranging from 0 to 100 (10 = warm (no pain); 20 = threshold pain; 100 = intolerable pain). The patients were instructed to rate the average intensity of axial neck pain in the *last month*.

### Postoperative

Postoperative neck pain intensity was also assessed using NRS from 0 to 100 at the 1-year follow-up telephonically. The patients were instructed to rate the average intensity of axial neck pain in the *last month*.

### ALFF calculation

For the ALFF analysis, a fast-Fourier transform was performed to convert the time series to the frequency domain. Subsequently, the square root of the power spectrum was calculated and averaged across 0.01–0.08 Hz to obtain the ALFF, and the resultant ALFF values were subsequently Z-scored. Therefore, we used zALFF in our current analyses.

### Analysis 1: Clinical data

We first Pearson correlation was performed to identify pairwise relationship(s) between all measured clinical features. Second, two-sample t tests were performed to reveal the differences in PVAQ score and pain threshold between DCM patient and HC, two-sample t tests were performed. Further, despite JOA score and preoperative/postoperative pain intensity were not investigated in healthy participants, the mean ± SD for both metrics were also illustrated. Third, we divided the DCM patients into postoperative axial pain (PAP) group and non-postoperative axial pain (nPAP) group based on the postoperative axial pain intensity using a cut-off value of 4 or more for NRS same as previous reports (Patients with postoperative pain intensity > 4 were included in PAP group) [7]. Furthermore, to rule out the possible confound of differences in severity of myelopathy, we also optimally match the JOA score between two group to avoid the possibility that differences we observed between nPAP and PAP group were due to the difference in severity of myelopathy using following procedures: (1) One target PAP patient was randomly selected, and the absolute differences for this target patient’s JOA score and the rest of the nPAP group were calculated; (2) This target patient was then matched with a patient whose JOA score was the closest to the target patient. If there were several nPAP patients whose JOA scores were the same as the target patient, one nPAP patient was then randomly selected; (3) These procedures were repeated until all PAP patients were paired. The un-paired nPAP patients were excluded for further analyses. Paired-t tests were performed to reveal the differences in clinical metrics (e.g., JOA score, PVAQ score, Pain threshold, Preoperative and postoperative pain intensity) between PAP group and nPAP group.

### Analysis 2: ALFF differences between DCM patients and HC

To reveal the differences in ALFF between DCM patients and healthy controls,voxel-wise two-sample t test was performed within a grey matter mask using SPM12 (http://www.fil.ion.ucl.ac.uk/spm) to explore the ALFF differences between DCM patients and healthy controls with age, gender, education years as covariates. Voxel-level *p* value ≤ 0.001 (significance threshold) was corrected for multiple comparisons using family-wise error correction at the cluster level, resulting in a corrected *p* ≤ 0.05. Subsequently, the resultant clusters were selected as masks to extract the mean ALFF for each cluster in DCM patients. Correlation analyses were performed to detect the association between ALFF alterations and clinical measurements in DCM patients, and Bonferroni correction was performed for multiple comparison correction.

### Analysis 3: ALFF differences between PAP and nPAP DCM patients

To investigate the possible neural mechanism for postoperative neck pain following posterior decompression surgery. Therefore, voxel-wise paired-t test was performed within a grey matter mask using SPM12 (http://www.fil.ion.ucl.ac.uk/spm) to explore the ALFF differences between PAP group and nPAP group (i.e., same as *analysis 1*) with age, gender, education years as covariates. Voxel-level *p* value ≤ 0.001 (significance threshold) was corrected for multiple comparisons using family-wise error correction at the cluster level, resulting in a corrected *p* ≤ 0.05.

### Analysis 4: Multi-variate classification—PAP vs. nPAP

To further test the utility of the ALFF (i.e., the mean within clusters obtained in *analysis 3*) for identifying PAP patients from nPAP patient, multi-variate pattern analysis (MVPA) was performed via support vector machine (SVM) using both clinical metrics and ALFF as features. Classification accuracy was assessed by a leave-one-out cross-validation procedure (LOOCV). The detailed procedure of LOOCV can be found in Additional files (*Leave-one-out-cross-validation procedure*).

A control analysis was also performed using only clinical metrics as features for PAP vs. nPAP classification. In this way, we can investigate whether ALFF could provide additional information for predicting the occurrence of postoperative neck pain in DCM patients. LOOCV and permutation test were also performed using the same procedures as described above. Furthermore, to test whether these two classification accuracies (e.g., using both clinical metrics and ALFF, using clinical metrics alone) were differ significantly, a permutation test was performed. The detailed procedure of permutation can be found in Additional files (*Permutation test*).

### Validation analysis

To further rule out the influence of head-motion as a potential confound, we conducted a validation analysis for revealing the differences in head-motion between DCM patients and HC; between PAP and nPAP DCM patients. Framewise displacement (FD) values, that quantifiably estimate head motion during scan, were calculated, averaged across all timepoints in all participants, and compared between groups. The FD value was calculated using 3 robust methods, Jenkinson method, Power method, and VanDijk method.

Moreover, to further make sure that any detected differences for ALFF between PAP and nPAP was determined by preoperative pain intensity, we also conducted a validation analysis to reveal the differences in clinical metrics and ALFF between PAP and nPAP by optimally matching the preoperative neck pain intensity between these two groups. The same approach as in *analysis 1 and 3* (i.e., the same procedures as matching the JOA score between two groups) was conducted and paired-t tests were performed to reveal the differences in clinical metrics (e.g., JOA score, PVAQ score, Pain threshold) between PAP group and nPAP group with age, gender, education years as covariates. In this way, there would be no significant difference for preoperative pain intensity between two groups, thus the observed ALFF differences between PAP and nPAP group would most likely to be associated with PAP rather than the reflection of the preoperative pain intensity.

## Results

### Demographic data

The demographic data of all participants are summarised in Table 1. There were no significant inter-group differences with regards to age, gender, or years of education (*p* ≤ 0.05).

**Table 1:** Demographic data of the two groups

	DCM (*n* = 62)	HC (*n* = 60)	*p* value
Age (years)	53.3 ± 7.38	53.4 ± 7.47	0.88
Gender (F/M)	31/31	30/30	1
Education (years)	12.1 ± 3.17	12.2 ± 3.23	0.47
JOA	11.4 ± 1.71		
Preoperative pain intensity	2.6 ± 1.22		
Postoperative pain intensity	3.8 ± 1.11		

*DCM* degenerative cervical myelopathy, *HC* healthy controls, *JOA* Japanese Orthopaedic Association

### Analysis 1: Relative to healthy controls (HC), degenerative cervical myelopathy (DCM) patients were more sensitive to pain

**Fig. 1:**
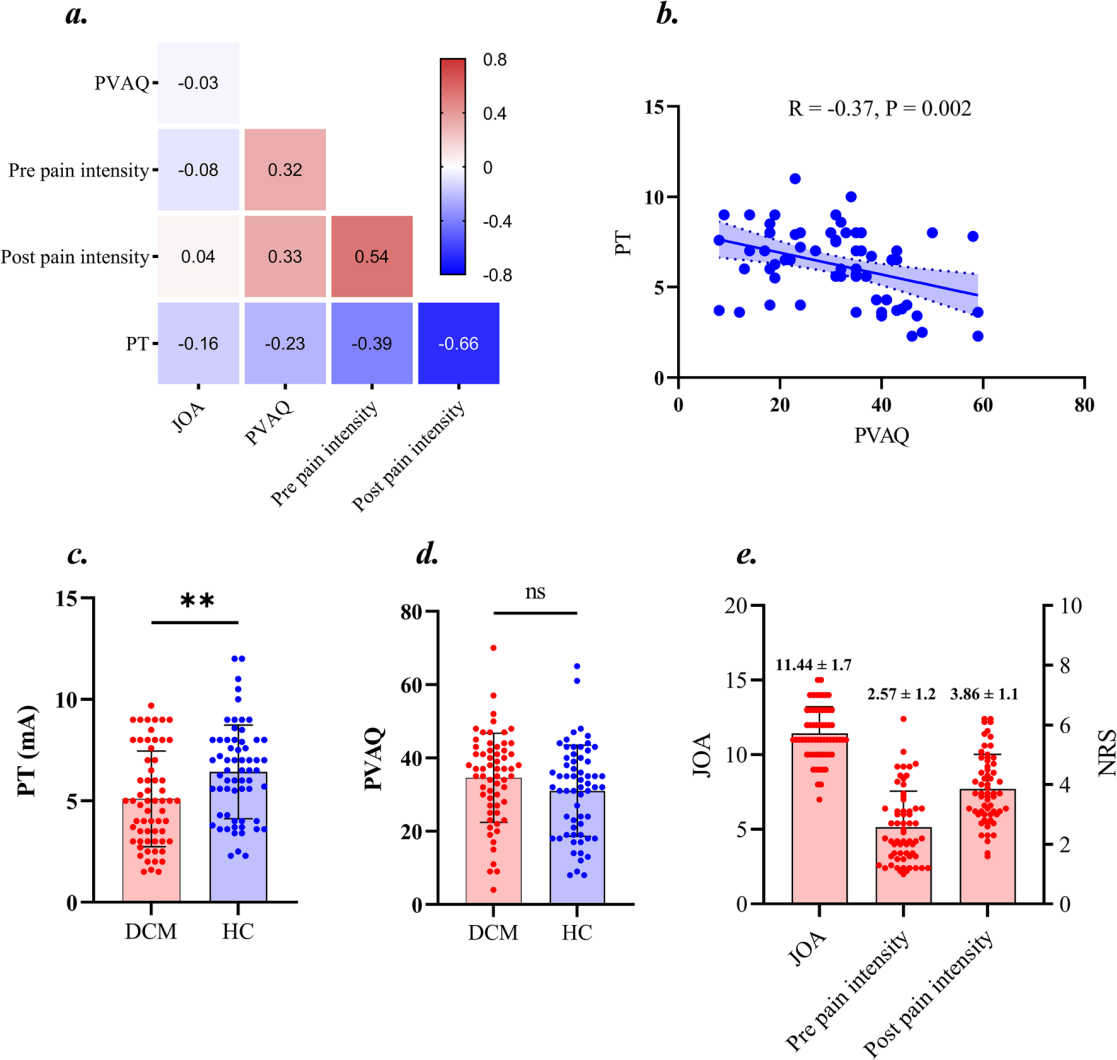
Analyses of clinical parameters. **a** The association among clinical metrics in Degenerative Cervical Myelopathy (DCM) patients; **b** The association between Pain Threshold (PT) and Pain Vigilance and Awareness Questionnaire (PVAQ) score; **c** The differences in PT between DCM patients and HC; **d** The differences in PVAQ between DCM patients and HC; **e** The mean±SD for Japanese Orthopedic Association (JOA) score, preoperative pain intensity and postoperative pain intensity in DCM patients. NRS: numerical rating scale

In DCM patients, we observed significant correlation between preoperative pain intensity and Pain Vigilance and Awareness Questionnaire (PVAQ) (*R* = 0.32, *p* = 0.005), between postoperative pain intensity and PVAQ score (*R* = 0.33, *p* = 0.004), between postoperative pain intensity and preoperative pain intensity (*R* = 0.54, *p* < 0.001), between preoperative pain intensity and Pain Threshold (PT) (*R* = −0.39, *p* = 0.001), between postoperative pain intensity and PT (*R* = −0.66, *p* < 0.001) Fig. 1a. In healthy participants, we observed a significant negative correlation between PVAQ score and PT (*R* = −0.37, *p* = 0.002) Fig. 1b. Further, compared with healthy participants, DCM patients exhibited significant decreased Pain Threshold (i.e., more sensitive to pain) Fig. 1c.

**Fig. 2:**
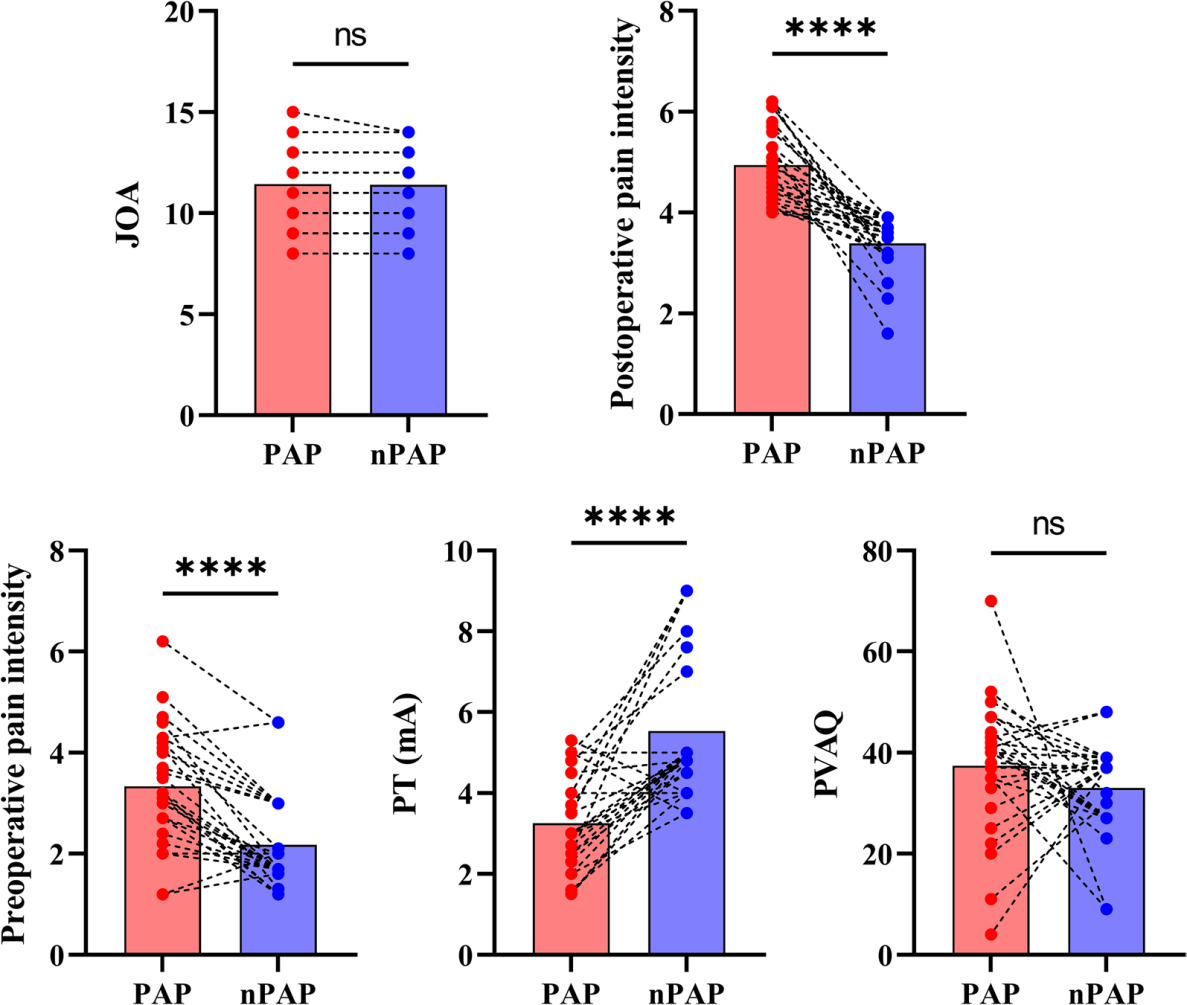
Clinical metrics differences between DCM patients have postoperative axial pain (PAP) and no postoperative axial pain (nPAP) while matching the Japanese Orthopedic Association score. PT: Pain Threshold; PVAQ: Pain Vigilance and Awareness Questionnaire; JOA: Japanese Orthopedic Association (JOA)

To further investigate the factors for postoperative axial pain in DCM patients following posterior decompression surgery, we divided the DCM patients into postoperative axial pain (PAP) group and non-postoperative axial pain (nPAP) group while controlling the effect of JOA score. Relative to nPAP group, PAP group exhibited significantly higher preoperative pain intensity along with lower pain threshold (i.e., more sensitive to pain) Fig. 2.

### Analysis 2: Compared to HC, DCM patients exhibited increased ALFF in Middle Cingulate Cortex (MCC) which was positively correlated with postoperative pain intensity and negatively correlated with pain threshold

**Fig. 3:**
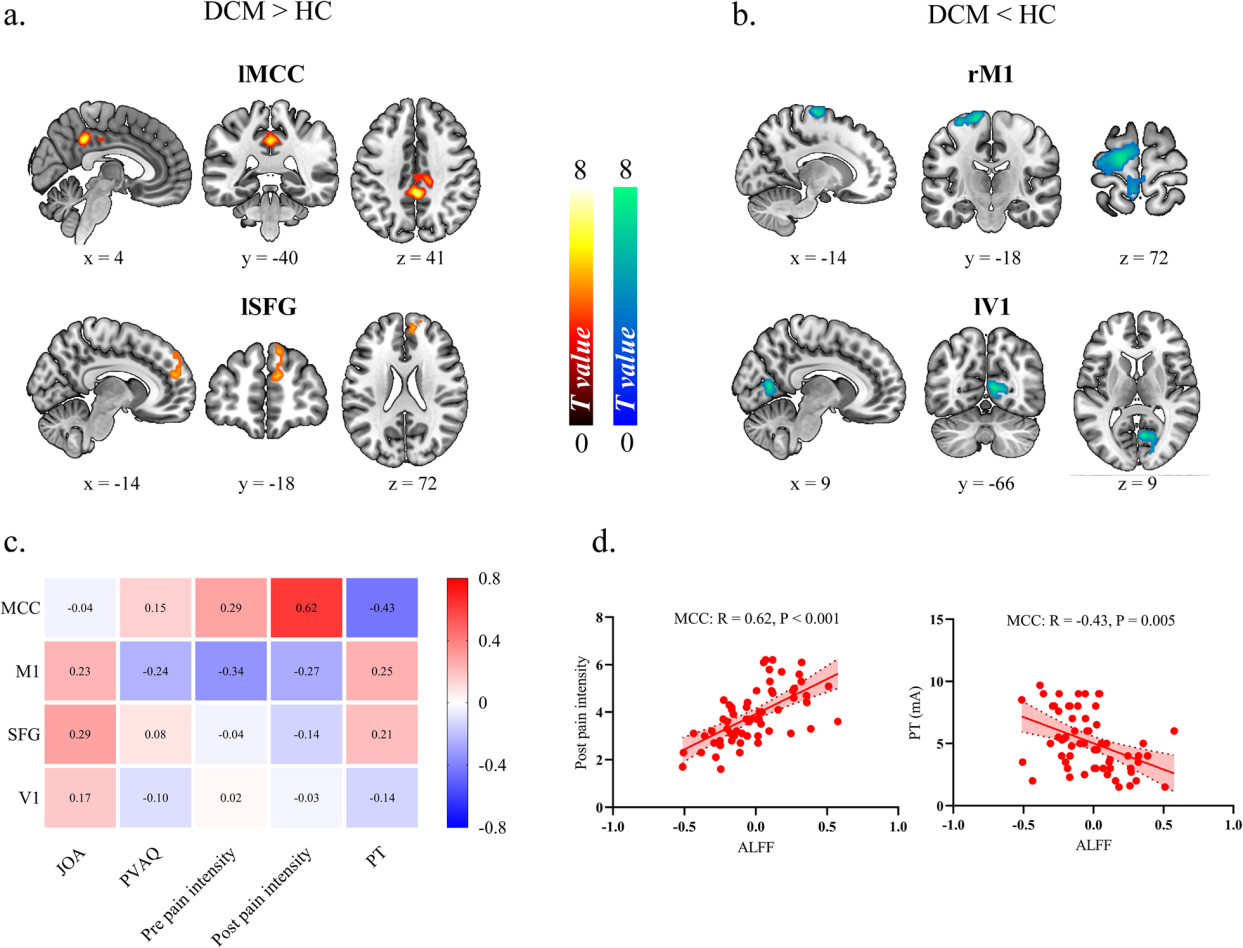
Analysis of Amplitude of Low Frequency Fluctuation (ALFF) alterations and its relationship to clinical metrics in Degenerative Cervical Myelopathy (DCM) patients. **a** The increased ALFF in DCM patients. lMCC: left Middle Cingulate Cortex; lSFG: left Superior Frontal Gyrus; **b** The decreased ALFF in DCM patients. rM1: right precentral gyrus; lV1: left calcarine gyrus. **c** The heat map for illustrating the correlation coefficients between brain alterations and clinical metrics in DCM patients. PT: Pain Threshold; PVAQ: Pain Vigilance and Awareness Questionnaire; JOA: Japanese Orthopedic Association (JOA). **d** The scatter plot for association between postoperative pain intensity and ALFF, between PT and ALFF within MCC in DCM patients

Relative to HC, DCM patients exhibited increased ALFF within left Middle Cingulate Cortex (lMCC) and left Superior Frontal Gyrus (lSFG) Fig. 3a. while decreased ALFF within right primary motor cortex (i.e., precentral gyrus, M1) and left primary visual cortex (i.e., calcarine, V1) Fig. 3b, Table 2, Additional file 1: Figure S1. We also observed a significant positive correlation between postoperative pain intensity and ALFF within MCC (*R* = 0.62, *p* < 0.001); a significant negative correlation between pain threshold and ALFF within MCC (*R* = −0.43, *p* = 0.005) Fig. 3c, d. No significant association was observed between clinical metrics and brain alteration within other brain regions.

**Table 2:** The detailed information for ALFF differences between Degenerative Cervical Myelopathy patients and healthy controls

Brain regions	Brodmann area	MNI coordinates			Peak intensity	Cluster size
Left mid-cingulate cortex	BA 23	0	−42	36	6.16	116
Right superior frontal gyrus	BA 9	12	57	30	5.81	68
Left precentral gyrus	BA 6	−15	−15	72	−7.23	309
Right calcarine	BA 17	9	−66	9	−5.32	113

### Analysis 3 and 4: MCC ALFF provide additional value for predicting the prevalence of postoperative axial pain in DCM patients

**Fig. 4:**
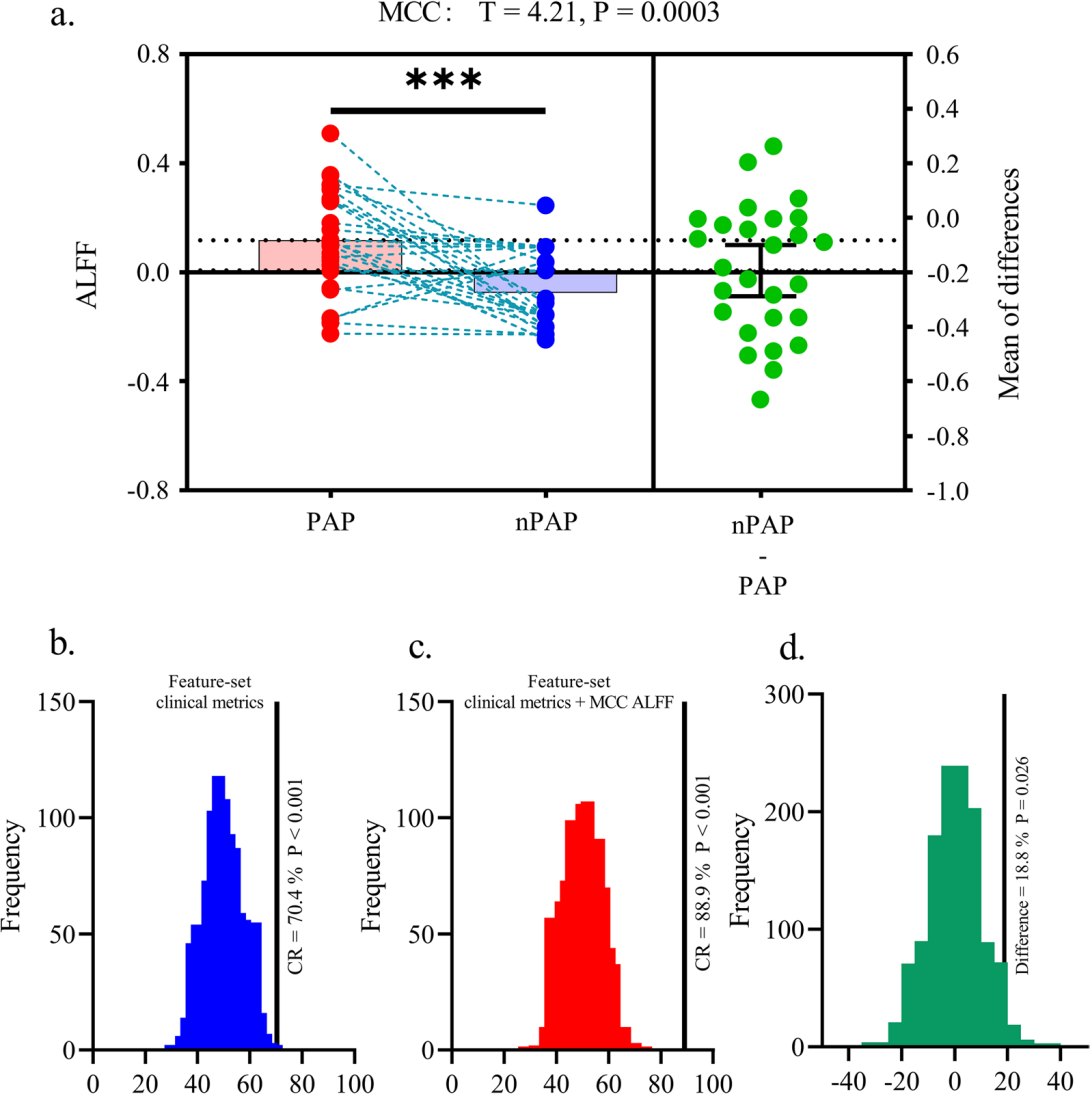
**a** The paired t test for revealing the Amplitude of Low Frequency Fluctuation (ALFF) differences between postoperative axial pain (PAP) group and no postoperative axial pain (nPAP) group in DCM patients. MCC: Middle Cingulate Gyrus. **b** The null distribution obtained from permutation test and performance of Support Vector Machine (SVM) model using only clinical metrics for PAP vs. nPAP classification. CR: Correct Rate. **c** The null distribution obtained from permutation test and performance of SVM model using clinical metrics combined with ALFF (i.e., within MCC) for PAP vs. nPAP classification. **d** Null distribution obtained from the permutation test to determine whether there is a significant difference between two models

In analysis 3, univariate paired-t test was performed to compare the ALFF between PAP and nPAP patients while controlling the effect of JOA, we observed that relative to nPAP, PAP group exhibited significant higher ALFF within MCC (*T* = 4.21, *p* = 0.0003, Fig. 4a, Table 3). Indicated that higher level of MCC ALFF was associated with a more intense postoperative axial pain in DCM patients. Subsequently, results for analysis 4 showed that the feature set including JOA, preoperative pain intensity, PVAQ, pain threshold along with age, gender, education years could successfully identify DCM patients with postoperative axial pain form patients without postoperative axial pain (Correct rate = 70.4, *p* = 0.002, Fig. 4b). After including the MCC ALFF to the feature set, the correct rate for PAP vs. nPAP classification increased to 88.9% (*p* < 0.001, Fig. 4c) and the difference between two model was significant (Difference = 18.8%, *p* = 0.026, Fig. 4d).

**Table 3:** The detailed information for ALFF differences between preoperative axial pain group and non-preoperative axial pain group

Brain regions	Brodmann area	MNI coordinates			Peak intensity	Cluster size
Left mid-cingulate cortex	BA 23	2	−43	31	5.78	106

### Analysis 5: Validation analyses

**Fig. 5:**
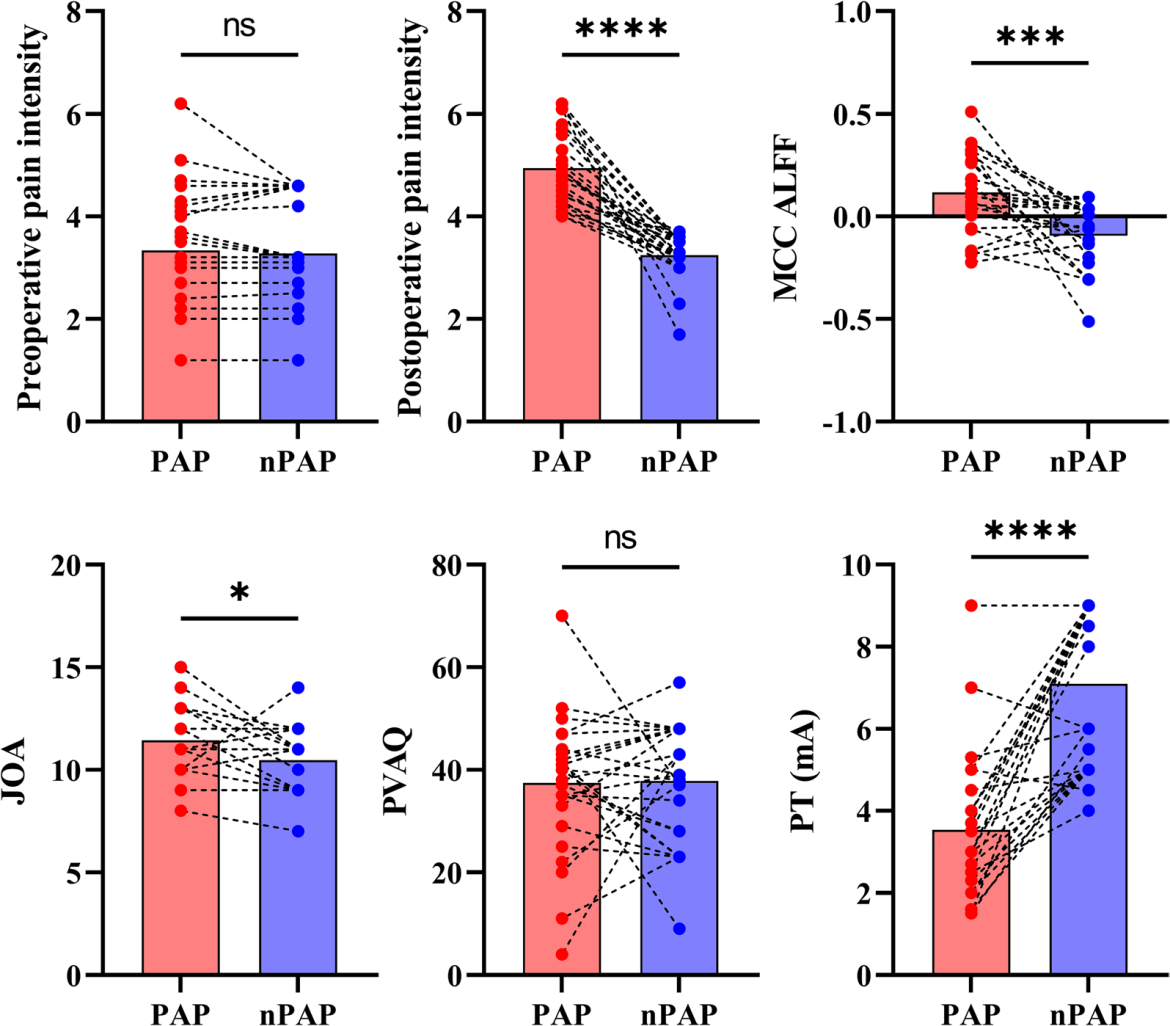
Clinical metrics differences between DCM patients with postoperative axial pain (PAP) and no postoperative axial pain (nPAP) while matching the preoperative pain intensity. PT: Pain Threshold; PVAQ: Pain Vigilance and Awareness Questionnaire; JOA: Japanese Orthopedic Association (JOA)

No significant difference for FD, which was measured by Jenkinson method, Power method, and VanDijk method, was observed (Additional file 1: Figures S2 and S3). We also found that after controlling the effect of preoperative pain intensity, PAP group still exhibited significant higher level of ALFF along with lower pain threshold (i.e., more sensitive to pain) relative to nPAP group (Fig. 5). These results were in line with our results in *analysis 1 and 3*, suggesting that the observed differences from analysis 1 and 3 were not affected by the preoperative pain intensity to a large extent.

## Discussion

In our current study, three major findings were observed: (1) Relative to healthy participants, Degenerative Cervical Myelopathy (DCM) patients exhibited lower threshold for pain; (2) and altered Middle Cingulate Cortex (MCC) function was associated with pain threshold which is also tightly correlated with the postoperative neck pain intensity; (3) Further, the ALFF of MCC provided additional value for predicting the occurrence of postoperative axial pain via machine learning analysis in DCM patients.

In comparison to healthy participants, DCM patients exhibited lower pain threshold; and patients with postoperative axial pain showed lower pain threshold preoperatively than those without.

In analysis 1, we found that the pain thresholds of DCM patients were significantly lower than healthy participants, and the pain thresholds were also lower in DCM patients with PAP than those without. This finding is in line with previous study conducted by Zhang et.al. in which they conducted quantitative sensory testing and revealed that patients with PAP have a lower pressure pain threshold and temporal summation (i.e., higher sensitivity to pain perception) than patients without PAP. Their findings indicated that preoperative endogenous pain modulation deficiency might be associated with axial pain after posterior cervical decompression [9]. It is not surprising that DCM patients developed abnormal pain modulation system, considering most of the patients experienced chronic pain that is associated with modifications of the central nervous system, such as central sensitization, which is responsible for alterations in pain sensitivity in acute and chronic pain situations [26–28]. We also found that preoperative pain threshold was negatively correlated with pre/post-operative pain intensity and preoperative pain intensity was positively correlated with postoperative pain intensity in DCM patients. These findings also supported the idea that DCM patients developed central sensitization following long-term axial pain which further aggravates or induce the postoperative axial pain.

### Altered MCC function was associated with preoperative pain threshold and PAP intensity in DCM patients

In analysis 2, we found that relative to healthy participants, DCM patients exhibited significantly higher ALFF within Middle Cingulate Cortex (MCC) and Superior Frontal Gyrus (SFG), and the MCC ALFF were correlated with both preoperative pain threshold and PAP intensity. MCC, which is frequently activated during acute pain, has been shown to be responded specifically to nociceptive input from subcortical brain regions. Additionally, chronic pain also causes grey matter changes in MCC, and such changes overlaps in various chronic pain condition indicating the structural alterations of MCC could well be the biological marker for chronic pain per se [29]. Further, Davis et.al. found that greater heat pain sensitivity (i.e., lower heat pain threshold) correlated with thickening in the mid-cingulate cortex, which indicated MCC is responsible for detecting and processing nociceptive input [30]. From the functional aspect, in addition, the MCC is an important component of the cingulate-insular pathway which gates and maintains nociceptive hypersensitivity in the absence of conditioned noxious stimuli and affects the impact of pain [31]. Taken together, our observed association between pain sensitivity and MCC ALFF support the hypothesis that continuous nociceptive input causes MCC cortical reorganisation which further induces hypersensitivity in chronic pain patients.

Furthermore, we also observed significant altered ALFF within SFG, M1 and V1. These results were in line with previous reports. Kaito et.al. conducted rs-fMRI and found that the ALFF within SFG and V1 were altered in DCM patients [32]. They concluded that these brain alterations were considered as the functional reorganisation following long-term chronic spinal cord injury. We also found that relative to healthy participants, DCM patients exhibited significantly lower ALFF within primary motor cortex (M1). M1, a key region in the sensorimotor network, is involved in a various of motor functions, such as motor planning, inhibition, coordination, movement, and so on [33, 34]. It has been shown that the ALFF within M1 was significantly higher in DCM patients than healthy controls, and was also tightly correlated with the fractional anisotropy value of C2 segment which reflects the severity of myelopathy [33]. Our previous study also illustrated the potential utility of M1 ALFF for predicting the prognosis of DCM patients following decompression surgery [35]. Our current finding was in line with the previous reports, indicating potential cortical reorganisation occurs in DCM [36–39].

### MCC ALFF provides additional value for PAP vs. nPAP classification

Posterior cervical decompression surgery is one of the most widely used surgical approaches, and increasing frequency of PAP after posterior decompression approach seriously affects the daily life of patients. Till now, there is still controversy about the causes of PAP and its related factors. Atsushi et al. showed that anterolithesis, current smoking, preoperative neck pain, etc. are influencing factors of axial pain after laminoplasty [7]. A systematic review summarises possible factors influencing axial pain after posterior surgery, including age, preoperative axial pain, different surgical techniques, and postoperative management [8]. It has been shown that about 40% of patients experienced axial pain after laminoplasty, but it occurred mostly in those who had preoperative axial pain [7, 8, 40]. Although multiple factors have been identified as causal factors in PAP, preoperative neck pain severity is the most commonly reported PAP marker in DCM patients, and our current results also confirmed this. Further, our univariate and multivariate analysis also identified the neural correlates of the PAP, which is associated with the pain sensitivity in DCM patients. It is also worth mentioning that despite we optimally matched the preoperative pain intensity between PAP and nPAP patients, PAP still exhibited significantly higher MCC ALFF than nPNP patients. This result indicated the independent contribution of MCC function in altered pain modulation pathway which related to hypersensitivity in PAP patients. As to the clinical implications of our findings, identifying patients with PAP could aid the clinicians to develop novel perioperative management to reduce or avoid such complication based on hypersensitivity in these patients. Preoperative analgetic use has been proved to be effective in reducing postoperative pain intensity for many other orthopedic surgeries [41–43]. Such perioperative preparation could reduce the central sensitization and thus relieve the pain following large trauma.

### Limitation

First, the main limitation is that our patients have all received medication treatment including non-steroid-anti-inflammatory drug, etc. This may affect our results to some extent. Therefore, future studies with DCM patients who are not on medication or who have a washout period from medication are needed to confirm our findings. Postoperative fMRI data was not collected due to the possibility of artifact and heating due to surgical implants. Even though it appears to be safe and other studies have collected data on postoperative fMRI data, the majority of our patients declined to cooperate after we informed them of potential harm (e.g., loss of surgical implants) associated with postoperative fMRI. Our current study only analysed ALFF alterations between patients and healthy controls, other resting-state fMRI metrics such as functional connectivity (FC), regional homogeneity (ReHo), functional connectivity strength (FCS), need further study. Socioeconomic status is a crucial factor affecting pain process between individuals, but was not collected and thus its possible association with pain perception could not be investigated in the present study.

## Conclusion

In conclusion, our findings suggest that the altered middle cingulate cortex function might be associated with preoperative pain hypersensitivity which aggravates postoperative axial pain in degenerative cervical myelopathy patients. This finding may inspire new therapeutic ideas for patients with preoperative axial pain.

## Supplementary Information

**Additional file 1:** Supplementary material.

## Data Availability

The data and codes used in this study can be availed upon reasonable request.

## Abbreviations

ALFF: Amplitude of Low Frequency Fluctuation
DCM: Degenerative Cervical Myelopathy
fMRI: Functional Magnetic Resonance Imaging
JOA: Japanese Orthopedic Association
LOOCV: Leave-one-out cross-validation procedure
PVAQ: Pain Vigilance and Awareness Questionnaire
PAP: Postoperative Axial Pain
SVM: Support Vector Machine
