# Supplementary material for: Preoperative pain hypersensitivity is associated with axial pain after posterior cervical spinal surgeries in degenerative cervical myelopathy patients: a preliminary resting-state fMRI study
Source: Insights Imaging. 2023 Jan 24;14:16. doi: 10.1186/s13244-022-01332-2 (PMC9871135; doi:10.1186/s13244-022-01332-2)
Supplement: Supplementary file 1 — Additional file 1: Supplementary material. [file 13244_2022_1332_MOESM1_ESM.pdf]

ELECTRONIC SUPPLEMENTARY MATERIAL

Preoperative pain hypersensitivity is associated with axial pain after posterior cervical spinal surgeries in degenerative cervical myelopathy patients: a preliminary resting-state fMRI study

Materials and methods

Subjects' inclusion criteria

Patients with Degenerative Cervical Myelopathy (DCM) were recruited according to the following criteria: (1) Evidence of myelopathy along the cervical spine (C3-C7) on cervical MRI; (2) Signs and symptoms of myelopathy correspond to MRI findings (e.g., sensorimotor deficits, bladder/bowel dysfunction, gait disturbance, etc.); (3) No prior history of cervical spinal surgery and agree to undergo decompression surgery (***e.g., laminoplasty***); (4) able to complete fMRI scan; and (5) no stenosis of the extracranial vertebral artery or the carotid artery following Doppler ultrasound examination: (6) No indication of any other neurological, psychiatric, ocular, or systemic diseases, including hypertension and diabetes; and (7) no history of alcohol or substance abuse.

The following criteria were used to recruit healthy subjects of similar age, gender, and education through advertisements (1) no evidence of spinal compression; (2) no other spinal or brain neurological disorders, or systemic disease; and (3) ability to complete fMRI studies; (4) No indication of any other neurological, psychiatric, ocular, or systemic diseases, including hypertension and diabetes; and (5) no history of alcohol or substance abuse.

fMRI data acquisition and preprocessing

Data acquisition

3T fMRI data were acquired using a MAGNETOM Prisma 3T MR scanner (Siemens, Erlangen, Germany) with a 64-channel phase-array head-neck coil. Sponge pads were performed to all participants to support the head for minimizing the head movement during the scan. All participants were clearly instructed to keep their eyes

closed and remain awake, while avoiding specific and strong thoughts. Furthermore, the head motion of the functional scan of each participant were calculated and the participants whose head motion were not within defined motion thresholds (i.e., translational or rotational motion parameters less than 2 mm or 2°) were required to underwent the fMRI scan again to minimize the effect of head motion.

BOLD signals were collected using prototype simultaneous multi-slices gradient echo echo-planar imaging (EPI) sequence using the following parameters: echo time (TE) = 30ms, repetition time (TR) = 800ms, field of view (FOV) = 222mm × 222mm, matrix = 74 × 74, in-plane resolution = 3mm × 3mm, flip angle (FA) = 54 degree, slice thickness = 3mm, gap = 0 mm, number of slices = 48, slice orientation = transversal, bandwidth = 1690 Hz/pixel, PAT (parallel acquisition technique) mode, slice acceleration factor = 4, phase encoding acceleration factor = 2. 450 images were taken in 6 min. A high-resolution 3D T1 structural image (2 inversion contrast magnetization prepared rapid gradient echo sequence, MP2RAGE) was also acquired using the following parameters: TR/TE = 4000ms/3.41ms, inversion times (TI1/TI2) = 700ms/2110ms, FA1/FA2 = 4 degree/5 degree, matrix = 256 × 240, FOV = 256mm × 240mm, number of slices = 192, in-plane resolution = 1mm × 1mm, slice thickness = 1mm, slice orientation = sagittal, total duration is 6 minutes, 42 seconds.

Data preprocessing

Functional MR data were preprocessed using the Data Processing Assistant for rs-fMRI (DPARSF; http://www.restfmri.net/forum/DPARSF) toolbox. The detailed preprocessing procedures were as following: (1) The first 10 volumes of each functional scan were excluded due to the acclimatization to the scanning environment and magnetization stabilization; (2) Motion correction were performed to remove the effect of head movement; (3) Functional images were co-registered to structural images and spatially normalized to the Montreal Neurological Institute template and each voxel was resampled to 3×3×3mm3; (4) The liner-drift, Friston-24 parameters, the mean global signal, the white matter signal, and CSF signal were extracted as covariates and regressed out to minimize nonneural signals; (5) Subsequently,

scrubbing for high motion timepoints was also performed; (6) Finally, a bandpass filter (0.01 ~ 0.08 Hz) was then applied to remove high-frequency noise effects; (7) resultant functional images were smoothed with an 8 mm full-width-half-maximum isotropic Gaussian kernel.

Leave-one-out-cross-validation procedure

In LOOCV: First, one data-point in the available dataset was held-out. Features were used to train a support vector machine model within the rest of the dataset and the model was then tested using the held-out test data-point, thereby yielding a predicted label for the test data-point. This procedure was repeated until each data-point was held out once as the test data-point. After that, an accuracy for this classification, which was determined as the proportion of accurate predictions out of total predictions were made, was used to evaluate the performance of the SVM model. The corresponding P-value was derived from the null distribution that was obtained from 1000 random permutation tests, by randomly shuffling the labels of the subjects in the training dataset, with the corresponding feature set. Specifically, the P-values were determined as a proportion of the number of permutations greater than or equal to the actual classification accuracy out of the total permutations. If none of the 1000 permutations reached the actual classification accuracy, the p-value was considered to be $P < 0.001$.

Permutation test

we used a permutation test method as following: (1) the difference between these two classification accuracies was calculated; (2) the labels of the subjects were randomly shuffled and divided into two groups. Subsequently, classification analyses were performed via SVM using clinical metrics combined with ALFF and just clinical metrics as features respectively; (3) the difference of the two classification accuracies obtained from step 2 was them calculated. These procedures were repeated 1000 times to obtain a null distribution and P-value as determined as a proportion of the number of permutations greater than or equal to the actual difference out of the total permutations. If none of the 1000 permutations reached the actual difference, the p-

value was considered to be P < 0. 001.

Sup-Figure 1

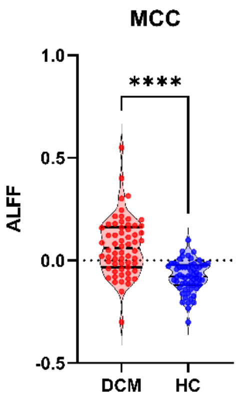

Amplitude of low frequency fluctuation (ALFF) differences between degenerative cervical myelopathy patients (DCM) and healthy controls (HC) within middle cingulate cortex (MCC). ****means $P < 0.0001$.

Sup-Figure 2

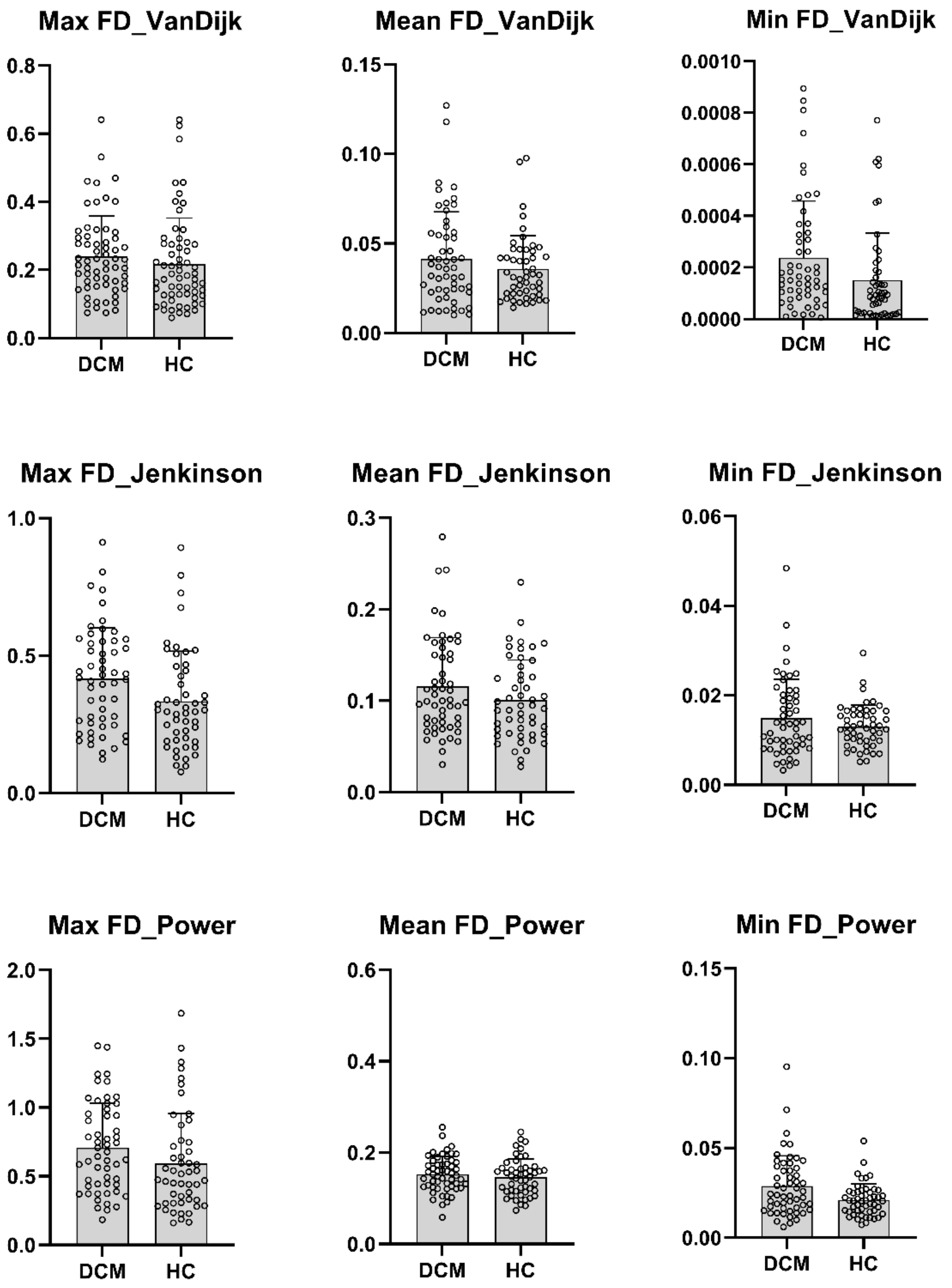

Max, mean and min FD value (i.e., FD Jenkinson, FD Power, FD VanDijk) differences between Degenerative Cervical Myelopathy (DCM) patients and healthy controls within each dataset.

Sup-Figure 3

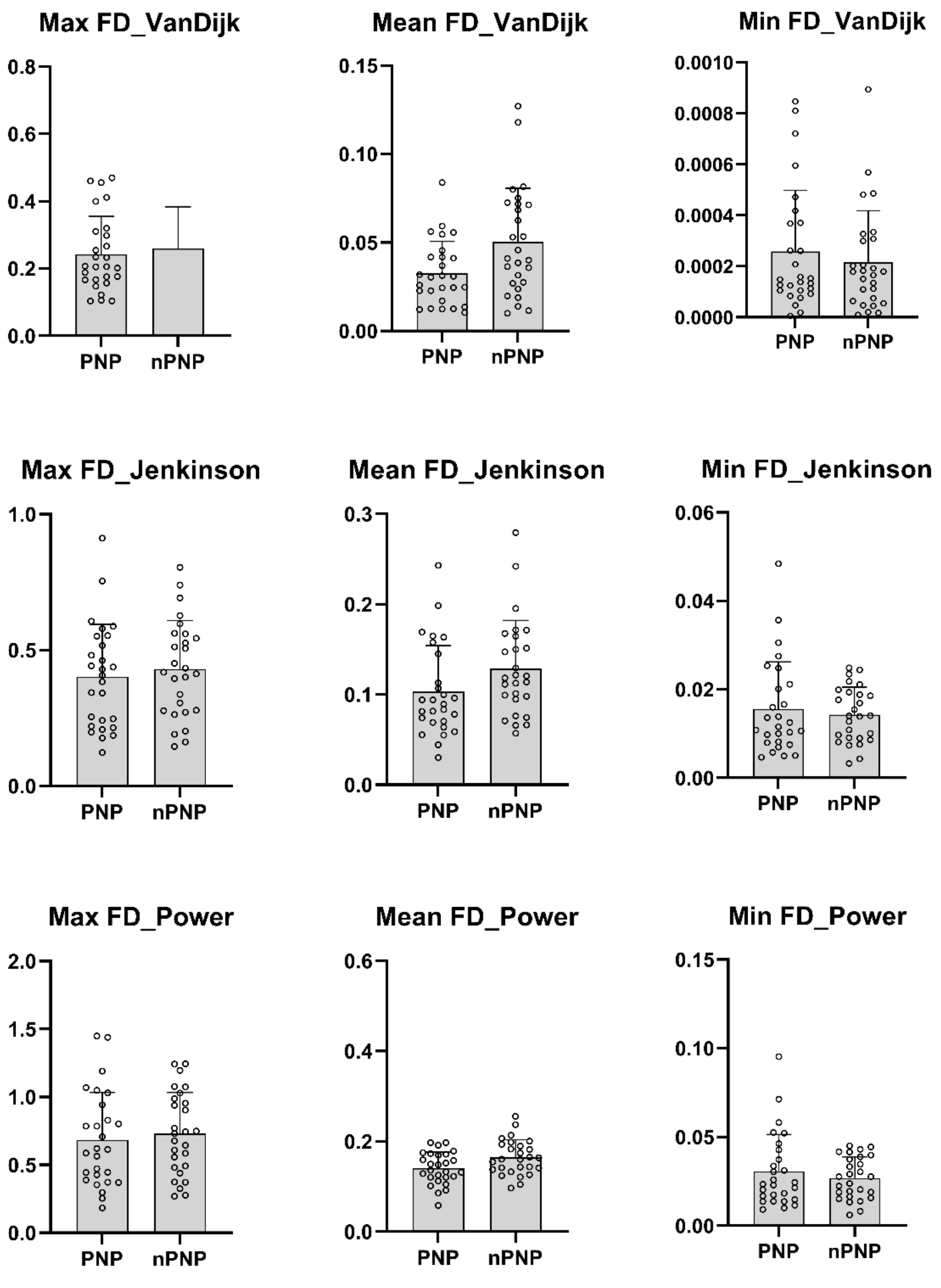

Max, mean and min FD value (i.e., FD Jenkinson, FD Power, FD VanDijk) differences between postoperative neck pain DCM patients and no-postoperative neck pain DCM patients after matching the Japanese Orthopedic Association score.
